# Needs, motivations, and identification with teaching: a comparative study of temporary part-time and tenure-track health science faculty in Iceland

**DOI:** 10.1186/s12909-019-1779-4

**Published:** 2019-09-11

**Authors:** Abigail Grover Snook, Asta B. Schram, Thorarinn Sveinsson, Brett D. Jones

**Affiliations:** 1 Faculty of Medicine, University of Iceland, 101 Reykjavik, Iceland; 2 Health Sciences School, University of Iceland, 101 Reykjavik, Iceland; 3 Physical Therapy Department, University of Iceland, 101 Reykjavik, Iceland; 4 Research Centre of Movement Science, 101 Reykjavik, Iceland; 5 Virginia Polytechnic Institute and State University, Blacksburg, Virginia USA

**Keywords:** Sessional faculty, Motivation, Needs, Faculty development, Identity, Connectedness, Pedagogy, Non-tenured, Adjunct, Part-time temporary, Self-determination theory

## Abstract

**Background:**

About 70% of teachers who instruct healthcare students are considered sessional (adjunct/temporary part-time) faculty and receive limited instruction in pedagogy. Sessional faculty may feel isolated and struggle with their teacher identity, and are often assumed to vary in their commitment, motivation, and ability to teach. However, research on teaching identity, motivations, and needs of sessional faculty is lacking. The aim of this study was to compare similarities and differences between sessional and tenure-track faculty across a health science school to guide faculty development for sessional faculty.

**Methods:**

We developed an online needs assessment survey, based on informal interviews and literature reviews. Seventy-eight tenure-track faculty and 160 sessional faculty completed the survey (37, 25% response rate, respectively). We used validated scales to assess intrinsic motivation, identified regulated motivation, and identification with teaching, as well as developed scales (perceived connectedness, motivated by appreciation to try new teaching method) and single items. All scales demonstrated good internal consistency. We compared sessional and tenure-track faculty using t-tests/chi-square values.

**Results:**

We found similarities between sessional and tenure-track faculty in intrinsic motivation, identified regulated motivation, and identification with teaching. However, sessional faculty perceived less department connectedness and were more motivated to improve instruction if shown appreciation for trying new teaching methods. Sessional faculty agreed more that they desired pedagogy instruction before starting to teach and that teachers should invest energy in improving their teaching. Admitting to less participation in activities to enhance teaching in the last year, sessional faculty were more interested in digital formats of faculty development.

**Conclusion:**

Our comparison suggested that sessional faculty value being a teacher as part of their self, similar to tenured faculty, but desired more appreciation for efforts to improve and perceived less connectedness to their university department than tenured faculty. They also preferred digital formats for pedagogy to improve accessibility, prior to and throughout their teaching career to support their development as teachers. Using this information as a guide, we provide suggestions for faculty development for sessional faculty. Supporting sessional faculty in the health sciences should improve the quality of teaching and positively affect student learning.

## Background

Faculty development (FD) has been shown to positively affect student learning [1]. Needs assessments are often used to take into consideration the needs of educators being served by FD. Traditionally, educators are asked about their perceived need for skills [2–5]. However, authors of the Best Evidence Medical Education (BEME) Guide on faculty development initiatives in the health sciences state that “the majority of (FD) interventions emphasized skill acquisition, often ignoring faculty members’ motivations for teaching, values, and professional identities” [6]. As FD interventions often challenge teachers to reflect on their teaching philosophy and consider modifying their current teaching practices, FD interventions need to build on the positive values and motivations that engage teachers, while providing the knowledge and skill that meets their perceived need [2, 7].

As students enter health sciences school, we assume that most are focused on becoming a good healthcare professional and not on becoming a good teacher. However, healthcare professionals may struggle with their identity as a teacher [8] as they find themselves teaching students as temporary part-time or, as they are referred to in Iceland and other parts of the world, sessional faculty (SF). Referred to by many other names across cultures and disciplines (e.g., adjunct, part-time, contingent, occasional, casual, non-tenured, community-based preceptors), SF are often appointed as non-tenure track and have a contractual, part-time relationship with their institution [9]. For this study, we defined SF as healthcare professionals who taught health science students directly in the classroom and/or clinic and were considered temporary, whereas tenure-track faculty (TF) were defined as all faculty hired for a permanent position.

SF are being called the “new faculty majority” [10]. They are gaining attention in research due to their prominence, with a U.S. study reporting 70% of all faculty are not hired on the tenure track [11]. Percentages of non-permanent faculty are difficult to report across Europe due to the heterogeneity of systems across and within countries [12], but the European University Institute reports that about 50% of faculty positions are fixed-term contracts in the United Kingdom [13]. Some researchers question whether the needs of SF are being met [9, 14, 15], especially in the areas of preparation for teaching and ongoing training. SF may vary in terms of their backgrounds, teaching abilities, and motivational levels for teaching students, which has led to questions about SF quality of instruction, loyalty, and the impact on student learning [16]. The Association of American Medical colleges reports that SF often feel that their status causes both the institution and their colleagues to doubt their commitment and work ethic [17], which may contribute to feelings of not being understood or appreciated. Weimer [18] states that SF may differ from TF with respect to certain needs, such as: 1) pedagogical – based on their limited exposure to teaching theories; 2) a sense of connectedness to the university, departmental colleagues/other SF; and 3) time for FD opportunities. Forbes et al. [19] writes that it is imperative that SF needs are assessed and addressed as a necessary first step to promote job satisfaction and quality in teaching among SF in nursing.

However, considering US research in the health sciences, SF are not well-represented in needs assessments and FD. In a report by the Alliance for Academic Internal Medicine, the authors suggest that little information is available regarding the experiences, satisfaction and engagement of SF [20]. Drowos et al. [21] reports that most FD for SF is based on informal conversations and teaching evaluations, rather than needs assessments. The conclusion then is that, without needs assessments, little is known about the unique professional needs, motivations to teach, and identity of SF [22], making it difficult to develop effective support for this ‘faculty majority’.

Although a few studies examine SF motivations to teach within the health sciences [23–26], most are qualitative, study a specific group within SF, and do not compare the results to TF. Therefore, we set out to answer the question: Do SF differ from TF with respect to their identification with teaching, motivation, connectedness with their department and faculty development needs across a health sciences school? We propose that a comparison of these two groups could suggest ways in which FD for SF should be different from FD for TF. Therefore, the aim of this study was to provide results that can guide FD for SF, by sending out a survey to both the TF and the SF in a health science school to compare measures of motivation, attitudes, values, and needs, highlighting similarities and differences.

## Methods

### Site, distribution, and participants

We performed this study at the Health Sciences School at the University of Iceland (HSS). HSS consists of six faculties: nursing, pharmacy, food science and nutrition, psychology, odontology, and medicine (which includes physical therapy, biomedical sciences and radiology). HSS has 212 TF and reportedly over 1000 SF [27], meaning SF comprise over 82% of teachers. To the best of our knowledge, no formal needs assessment of HSS teachers, whether SF or TF, has ever been conducted.

We obtained TF email addresses through online resources for HSS as well as distributions across disciplines and gender. No centralized list of health science SF email addresses was available from the university, so a list was generated through various resources. We coded SF and TF email addresses for incentive awards ($20 gift cards) and only the primary researcher had access to the codes to preserve anonymity. For both the pilot and the main study, the primary researcher sent the invitation to participate from the HSS faculty developer (second author of this paper) email, explaining its purpose as an anonymous needs assessment and encouraging participation, while including the code and a link to the online survey. A few departments agreed to send out general encouragements to their faculty to participate. Once the participant entered the online survey, the purpose of the anonymous survey was again explained, the participant was given the primary authors’ contact information for any questions, and it was explained that the results would be utilized for publications with all self-identifying information removed. The primary author sent reminders after two weeks to teachers who had not participated. Participation in the survey served as consent for participation in the study.

### Survey development

Following the AMEE Guide for developing questionnaires [28], we performed a literature review of theories related to faculty needs assessment and a review of recent surveys [2, 3, 5, 19, 29, 30], which was synthesized with teacher interviews into a survey. We included previously validated scales that assessed the constructs described here. *Identification with teaching* (ID) is a 4-item scale, adapted from engineering, to evaluate identification with a profession [31]. ID is a measure of the extent to which a teacher values their role and performance in teaching as an important part of self [32]. *Intrinsic motivation* (IM) is a 4-item scale used as part of the Physician Motivation Teaching Questionnaire (PMTQ) [33]. IM is a construct within self-determination theory (SDT) and involves the highest form of self-regulation [34], where actions are done out of pure interest or joy. *Identified regulated motivation* (IR) is a 3-item scale, also part of the PMTQ [33]. IR, also part of SDT, is considered close to IN, with actions based on personal values and beliefs.

We also developed two scales based on the literature review: [1] a 3-item scale of teachers’ perceived connectedness with their department/colleagues (CO) and [2] a 4-item scale of motivations to try a new teaching method by forms of appreciation (AP) (acknowledgment, financial compensation, supervisor feedback, improved student evaluations). We also included items that measured perceived need for more pedagogy before starting to teach, attitudes towards teachers’ responsibilities to improve teaching, participation in faculty development activities in the last year, and FD format preferences. We chose to have our participants rate most items on a 6-point Likert scale (1- “strongly disagree”, 2- “disagree”, 3- “somewhat disagree”, 4- “somewhat agree”, 5- “agree”, and 6- “strongly agree”) as we were interested in the strength of their agreement/disagreement. We did give an option of “choose not to answer”. For preferred FD formats, we utilized a 5-point Likert scale (1– “never”; 2– “very unlikely”; 3– “unlikely”; 4– “likely”; 5– “very likely”). A copy of the entire survey in English is available upon request from the primary author.

We utilized suggested guidelines [35] for the adaptation of the survey to Icelandic, which included translation by a bi-lingual expert into Icelandic, synthesis, back-translation by a second bi-lingual expert into English, review by an expert committee, and pilot-testing with review. Thirty-two TF and 48 SF from HSS participated in the online pilot testing conducted a month prior to general administration of the survey. Icelandic translation of validated scales showed similar internal consistency to previously reported measures with all scales showing good internal consistency with Cronbach’s alpha (α) [36], as listed in Table 1 along with the complete list of items for ID, IR, IM, CO, and AP scales.

**Table 1:** Scales - internal reliability and items

α, α from literature	Scale name	Scale items
.80, .84 [31]	Identification with teaching (ID)	Success in teaching is very valuable to me
		It matters to me how well I do with my teaching
		Being good at teaching is an important part of who I am
		Doing well as a teacher is very important to me
.86, .82 [33]	Intrinsic motivation (IM)	I enjoy teaching most of the time
		I look forward to my next teaching most of the time
		During teaching, I am completely in my element
		Teaching enriches my job
.80, .65 [33]	Identified regulated motivation (IR) “I teach because…”	I find the contents of my lesson important
		I am convinced that it is a healthcare professional’s duty to pass on his/her knowledge
		it’s important for me to make my contribution to students becoming good healthcare professionals in the future
0.78	Perceived connectedness with department (CO)	Department members frequently share teaching methods they have found successful
		I feel connected to my department colleagues
		I have specific department colleagues whom I would look to for help if I wanted to improve my teaching methods
0.76	Motivated by appreciation (AP) “I would be motivated to try a new teaching method…”	if I was financially rewarded for attending course and workshops on enhancing my teaching
		if I received feedback from other teachers or my supervisor on my teaching
		if it improved my ratings on student evaluations
		if I was shown appreciation for enhancing my teaching methods

### Ethical considerations

We submitted the details of the project to The National BioEthics Committee. In response, they indicated there was no need for their approval given the nature of the data being anonymous opinions of faculty. We announced the project to the Icelandic National Data Protection Authority who publicized the project as per Icelandic regulations. This research was part of the primary author’s doctoral study that was approved by the University of Iceland School of Health Sciences. The two primary researchers who sent the invitation emails had no position of authority over the participants and participation in the survey was voluntary and anonymous.

### Data analysis

Pilot testing identified no single item measures that were problematic due to the translation process; therefore, we added the pilot data to the main data collected for full analysis. For all statistical analyses of final data, we utilized SAS 9.4 (SAS Institute Inc., Cary, North Carolina, USA). We calculated a total scale score by summing the scale items, and then divided the total by the number of items to determine the average scale score. Independent-sample t-tests were used to identify similarities and significant differences between SF and TF for the five scales.

To eliminate cells with values less than five in the chi-square test, we combined the “strongly disagree”, “disagree”, “somewhat disagree”, and “somewhat agree” statements into one category. For preferred FD format, we combined “likely” and “very likely” scores into one category and “never”, “very unlikely”, and “unlikely” scores into another category. We then calculated chi-square values to identify differences between SF and TF for single item measures. We performed a two-way ANOVA on all significant scales and single item measures (other than preferred FD format) with the teacher type (SF or TF) and levels of [1] gender (male or female), [2] age group (< 40, 40–52, or 53+), and [3] discipline (nursing, medicine, or physical therapy) to test for interactions between them.

## Results

Between the pilot testing and general administration of the survey, we emailed 863 invitations to participate to TF (*n* = 212) and SF (*n* = 651). Although 298 faculty members entered the survey, we collected demographic information (i.e., gender, age range, faculty discipline within HSS, SF or TF, and number of classroom teaching hours for SF) at the conclusion of the survey and we discarded data for the comparison if demographic information was not complete. Seventy-eight TF and 160 SF (or 238 faculty in total) completed the survey and demographic information for a response rate of 37 and 25%, respectively. SF who teach in the classroom averaged 40 h a year of teaching (range 2–813 h, median = 16 h), or 1.3 h/week. A comparison of the demographic information of our TF and SF is presented in Table 2. We saw in our comparison of the TF survey respondents to the TF reported by the university that the survey respondents were an approximate representation of the disciplines at HSS but had a higher percentage of females. We could not perform the same comparison for SF as the information on SF was limited to their email addresses. In Table 2, we also compared the SF survey respondents to TF survey respondents, indicating that the SF group had a higher percentage of females and medicine faculty (includes physical therapy, biomedical sciences, and radiology), and was younger. Due to these differences, we performed a two-way analysis of variance, which indicated that interaction effects of teacher type with gender, age group and discipline were non-significant for scales and items (discussed below).

**Table 2:** Participant Characteristics

	TF		SF	
	R (%F)	S (%F)	R	S (%F)
# of participants	212 (45)	78 (62)	651	160 (71)
% Med	56 (36)	54 (42)	*	66 (64)
% RN	15 (87)	19 (87)	*	22 (94)
% N&FS	6 (46)	8 (67)	*	1 (100)
% Odont	9 (25)	6 (40)	*	2 (50)
% Pharm	6 (57)	5 (25)	*	4 (66)
% Psych	8 (50)	8 (67)	*	5 (40)
% > 52 years old	*	54	*	38

TF = tenured faculty; SF = sessional faculty;

R - reported by university; S - survey respondents; %F – percent female;

Med - faculty of medicine (includes physical therapy, biomedical sciences, radiology); RN - faculty of nursing; N&FS – faculty of nutrition and food science; Odont – odontology; Pharm – pharmacy; Psych - psychology; *information not known

A comparison between SF and TF across our motivation (IM, IR), identity (ID), connectedness (CO), and appreciation (AP) scales, using independent t-tests is presented in Table 3. We saw similarities and differences between SF and TF. The *p* values indicated that there were no significant differences between TF and SF with respect to intrinsic motivation (IM), identified regulated motivation (IR), and identification with teaching (ID), but inter-individual variation did exist as seen in measures of standard deviation (SD). In contrast, as evidenced by the *p* values, there were significant differences with SF demonstrating significantly less connectedness (CO) and more motivation to try a new teaching method if shown appreciation (AP) than TF. Our developed scales (CO, OP) had higher inter-individual variation (SD), lower average scores (M), and more participants who chose not to answer, as indicated by the degrees of freedom (DF), when compared to our validated scales (ID, IM, IR).

**Table 3:** Scale Comparisons Between Tenured and Sessional Faculty

	TF		SF				
Scale	M	SD	M	SD	DF	t	p
IM	5.1	0.7	5.0	0.8	234	1.43	0.23
IR	5.5	0.6	5.5	0.5	236	0.1	0.75
ID	5.5	0.5	5.5	0.6	232	0.56	0.45
CO	3.8	1.2	3.2	1.2	201	3.36	< .001
AP	4.2	1.1	4.6	0.9	209	6.07	0.01

M - average score; SD - standard deviation; DF - degrees of freedom;

TF - tenured faculty; SF – sessional faculty;

IM - intrinsic motivation; IR - identified regulated motivation; ID - identification with teaching; CO - perceived connectedness; AP - motivated to improve by appreciation

Response options included: 1-strongly disagree; 2-disagree; 3-somewhat disagree; 4-somewhat agree; 5-agree; 6-strongly agree

Table analysis with chi-square testing is provided in Table 4 for single items that evaluated attitudes towards and participation in activities to improve teaching. Again, we saw some differences between SF and TF as indicated by significant *p* values. SF significantly agreed more than TF that they would have liked more pedagogy before starting to teach, with 45% agreeing strongly with this statement. The *p* values also indicated that SF agreed more that it is part of a teacher’s responsibility to invest time and energy to improve teaching. With the largest p value difference, SF admitted more to not participating in activities in the last year to enhance teaching (44%) compared to 13% of TF.

**Table 4:** Item Comparisons

			DSA	A	SA	DF	SS	Chi-square	p
I would have liked more pedagogy before I started teaching	TF	Count	31	27	20	2	235	7.92	0.019
		% within teacher type	40%	35%	25%				
	SF	Count	46	41	70				
		% within teacher type	29%	26%	45%				
It is part of a teacher’s responsibility to invest time and energy to improve teaching	TF	Count	36	22	17	2	222	7.31	0.026
		% within teacher type	48%	29%	23%				
	SF	Count	48	70	29				
		% within teacher type	33%	47%	20%				
			3+	1 or 2	0				
The number of times I participated in activities that developed my teaching methods in last year.	TF	Count	33	35	10	2	238	33.96	<.0001
		% within teacher type	42%	45%	13%				
	SF	Count	22	67	71				
		% within teacher type	14%	42%	44%				

DSA- strongly disagree, disagree, somewhat disagree, somewhat agree; A - agree; SA - strongly agree;

TF - tenured faculty; SF - sessional faculty

DF - degrees of freedom; SS - sample size; p - significance level

Table analysis and chi-square testing for significant preferred FD formats is presented in Table 5. SF preferred distance learning, hybrid learning, videoconferencing, and social networks when compared to TF, with distance learning being the most popular (67%). The largest differences (*p* < .001) were seen with the distance learning and social networks formats. Other formats that were asked about on the survey (e.g., workshops, consultations, in-person discussion groups) were not significant between groups.

**Table 5:** How likely are you to participate in FD with following formats?

			UL	LL	DF	SS	Chi-square	p
Distance learning	TF	Count	50	28	1	238	20.5	<.001
		% within teacher type	64%	36%				
	SF	Count	53	107				
		% within teacher type	33%	67%				
Hybrid learning	TF	Count	41	37	1	238	4.5	0.035
		% within teacher type	53%	47%				
	SF	Count	61	99				
		% within teacher type	38%	62%				
Videoconference	TF	Count	51	27	1	238	5.4	0.02
		% within teacher type	65%	35%				
	SF	Count	79	81				
		% within teacher type	49%	51%				
Social networks	TF	Count	55	23	1	238	14.4	<.001
		% within teacher type	71%	29%				
	SF	Count	71	89				
		% within teacher type	44%	56%				

FD - Faculty Development; TF - tenured faculty; SF - sessional faculty

UL - never, very unlikely, and unlikely; LL - likely, very likely

DF - degrees of freedom; SS - sample size; p - significance

## Discussion

To the best of our knowledge, this is the first quantitative survey across an entire school of health sciences comparing the motivations, attitudes, values, and needs of both SF and TF. Our response rate is modest, possibly due to the survey length, but comparable to other needs assessments [2, 3, 29]. It is difficult to determine how representative our results are for the actual SF population since the university lacks information on this group, a problem also experienced in other studies [37, 38]. However, our results have value in illuminating the needs of an under-represented group of teachers who play an important role in health science education. We believe the results contribute to a better understanding of the similar and different motivations and needs of both TF and SF at a health sciences school and can help universities support all health science faculty.

### Similarities between SF and TF

#### Intrinsic/identified regulated motivation and identification with teaching

The results from this study suggest that there is no difference in intrinsic motivation between SF and TF (Table 3). Both TF and SF agree that teaching is enjoyable and personally fulfilling, which is encouraging to see. This is similar to a report of SF in nursing where participants describe their work as ‘positive’ and ‘rewarding’ [39] and in qualitative studies with SF hospital physicians who describe ‘the joy of teaching itself’ [24, 25]. Results from Steinert and Macdonald [23] “suggest that we should acknowledge our teachers, nurture their inherent desire to teach, and make the joy of teaching more visible.” According to our results, the environment provided at HSS seems to be supporting both SF and TF interest and enjoyment of teaching fairly well.

The results also suggest that there is no difference in identified regulated motivation between SF and TF (Table 3). IR includes the values and beliefs integrated into a teacher that become his/her reasons to teach. Altruistic statements (e.g., “teaching is a healthcare professional’s duty”) are strong in the health sciences and are mentioned often in the literature among all types of faculty [23–26]. As these values are similar and just as strong in SF, our results contradict suggestions that SF are less motivated and committed to passing on their knowledge to students [17] and suggest that SF have strong professional values regarding teaching.

Deci and Ryan (34, pg. 182), in describing SDT, state that autonomous motivation is “both intrinsic motivation and the types of extrinsic motivation in which people have identified with an activity’s value and ideally will have integrated it into their sense of self”. Therefore, the combination of similar scores on both IM and IR suggest that SF and TF have similar autonomous motivation for teaching. Supporting autonomous motivation maximizes functioning and well-being in both students and faculty [40], so we suggest it would be advantageous to encourage and celebrate IR and IM in all teachers.

The results also suggest that there is no difference in identification with teaching between TF and SF (Table 3). ID is a representation of how much a person values their role/performance in teaching, as a part of their self, as part of their identity [41]. We consider this a somewhat surprising result as a common assumption is that SF do not value being a teacher as much as TF. This is assumed because teaching is most often a secondary profession to their work as a healthcare professional, as evidenced by our sample which has an average classroom teaching of 1.3 h per week. The fact that TF and SF report similar high values for ID seems to contradict assumptions that SF identify less with being a teacher and are less motivated and committed to teaching [16, 17] and indicate that they identify themselves as teachers similar to TF.

### Differences between SF and TF

#### Less connectedness to department/colleagues

Our results confirm the conclusions of many qualitative studies that found that SF perceive a lack of connectedness to their university departments/colleagues [9, 16, 17, 22, 39, 42] (Table 3). In SDT, connectedness is similar to relatedness and is considered a fundamental need for optimal functioning and growth [43]. Common descriptions of life as a SF in the literature include words like ‘isolated’, ‘not belonging’, ‘limited contact with faculty’, ‘lack of institutional engagement’, ‘excluded’, ‘invisible’ and ‘outsiders’ [19, 22]. Buch et al. [9] report that the overwhelming challenge mentioned by SF is ‘the sense of isolation and disconnectedness from their departments and colleagues’. SF are also excluded from course development and curriculum renewal, further removing them from essential planning functions at the university [39]. Not only do SF experience less connectedness but a longitudinal study suggests these feelings ultimately increase over time and affect faculty identity negatively [44]. Research studies are starting to appear in the literature on programs designed to address SF needs for more connectedness [9, 16, 42] but there is still a lack of in-depth evaluations regarding program outcomes [14]. However, increasing a sense of connectedness for SF is hypothesized to improve retention of effective SF [19] and is associated with improved loyalty and satisfaction [45] and less reports of isolation by SF [9], which may improve teaching effectiveness and student outcomes.

#### Motivated by appreciation

Our study results also suggest that SF would be more motivated than TF to reflect on their teaching or try a new teaching method if they are shown forms of appreciation (Table 3). These forms of appreciation include general recognition, feedback from supervisors, financial compensation, and improved student evaluations. Although all are considered extrinsic forms of motivation by SDT, general recognition, feedback from supervisors, and improved student evaluations can be considered either identified regulation or introjected regulation, depending on whether the teacher is motivated by internalized values (identified regulation) or to avoid guilt or enhance pride (introjected regulation) [43]. They are, therefore, considered more supportive of autonomy than financial compensation, considered to be a form of external regulation by SDT [43].

All health educators alike cite a need for recognition of high-quality teaching [4, 46] and a sense of appreciation from others has been recognized as important to identity development as a health science educator [8]. However, a common complaint of SF is not feeling appreciated for what they contribute [4, 42, 47]. Hoyt [45] reports low ratings of perceived recognition among SF, even though it is identified as a primary motivator for loyalty and satisfaction. Similarly, SF complain of a lack of assessment of and feedback on their teaching, which can be perceived as a lack of caring by the department/institution [22]. However, van den Berg et al. [29] reports that ‘feedback on my teaching performance’ is the strongest predictor of engagement in teaching faculty at a university medical center. Therefore, it appears that feedback regarding teaching and appreciation of good teaching will benefit SF. Better student evaluations will presumably improve any teacher’s sense of appreciation, although some argue that overemphasis on student evaluations, as is seen in assessments of SF [22], can inhibit diversification of methods by teachers due to fear of receiving lower student evaluation grades and wasting students’ time [48]. Results are not as clear about the need for financial compensation but more recent research shows that the loyalty of SF is partially predicted by honorariums [9, 45].

#### More pedagogical training

Our results indicate that SF desired more training in pedagogy before starting to teach, with over 71% of SF agreeing/strongly agreeing with this statement (Table 4). This supports results from qualitative studies that suggest that SF in the health sciences receive little, if any, training in teaching methods, and desire more training [3, 9, 49, 50]. An important question persists in the health science professions: How can we expect our teachers to be effective and competent teachers if we rarely train them in teaching? A sense of competence has also been identified as essential for health science educator identity [8] and optimal functioning and growth according to SDT [43], and a lack of confidence in teaching ability has been identified as a barrier to teaching medical students [49].

#### Responsibility to improve but not attending FD

More SF than TF agree/strongly agree that it is part of their responsibility as a teacher to invest time and energy to improve their teaching, while 56% of SF state that they attended at least one activity to enhance their teaching in the last year, as compared to 87% of TF (Table 4). Little literature on SF attendance at FD activities exists. Buch et al. [9] reports 67% of SF participated in at least one activity in FD in the last year and Hoyt [45] reports 69% of SF agreeing/strongly agreeing that they had enhanced their teaching in the last year. Both studies report somewhat higher percentages than the current study report of 56%. Part of SF support could include FD that is available in various formats favored by SF (e.g., distance and hybrid learning), as supported by results in Table 5. In support of all these conclusions, a qualitative analysis by Buch et al. [9] identifies the following encouragers for SF to attend FD: 1) convenient time; 2) digital/online formats unless there is a social aspect; 3) increasing SF awareness of FD workshops and offerings; 4) offering workshops that are relevant and have proven benefits to students; and 5) incentives (appreciation, financial compensation) to participate.

### Implications

We suggest that useful information was obtained about SF and TF through the current survey. Our results support the fact that SF have similar motivations and values to TF and confirms that SF put their value of self in the role and performance of being a good teacher, even if it may be a secondary occupation. We suggest that the conclusion from these results should be that SF should not be “marginalized” or neglected by their institution/department because of the presumption that they are not as motivated or committed to being a good teacher.

The results also reinforce the idea that SF have a need for connectedness that should be addressed by FD, departments, and the university. There are many suggestions in the literature of what this could entail. A few universities have developed unique SF orientations [9, 14, 50] and required courses that are compensated [21], so that new SF have a basic pedagogical background before they start teaching. Another important idea is a centralized office for SF within the department or university [15, 17] with updated contact information. This may improve awareness of FD among SF and the office can develop resources specific to SF, such as orientations, webpages, evidence-based pedagogical strategies, classroom observations, and mentoring [9, 45, 50]. Within the department, SF can be invited to meetings, be a voice in department and curricular decisions, and meet with staff, department chairs, and other SF for both professional and social reasons [19, 39]. Other ideas that have been found to be successful are faculty learning communities and book clubs for SF [9, 16, 51]. Focus groups and interviews with the SF group of interest can identify and guide solutions that work best for that population.

One way to address the inconsistency between SF desire/value of FD and actual participation is to make FD offerings convenient to SF (multiple times including after-work hours and/or through various innovative digital formats) [9, 21]. Recognition of good teaching and the efforts made to become a better teacher are essential, and pedagogical training for SF that is reimbursed or somehow acknowledged should become the standard [21], and be seen as an investment in teachers and students. FD has been shown to improve teacher identity by improving confidence in teaching ability, increasing relatedness to other faculty, and increasing credibility and legitimacy as educators [8], as well as positively affecting student learning [1]. Therefore, FD should be encouraged and accessible for all health science faculty.

### Limitations and suggestions for future research

We acknowledge that there are limitations to the study. First, the survey was administered to faculty in only one health science school. Therefore, some of the results obtained could reflect specific issues within this health science school and not be generalizable to all health science schools. However, results from the literature are consistent with the results shown from the study, indicating that the faculty at HSS in Iceland are similar to faculty at other schools with respect to at least some of their beliefs. Second, we cannot say with certainty that the results of this sample are representative of the whole population of teachers at HSS given the response rate and the fact that there was a high representation of female faculty in our respondent population, a common problem in survey research. In future research, the response rate might be better if a shorter measure or a specific tool could be created from the current study survey, as our respondents’ completion rate indicated there was some survey fatigue (298 entered the survey but 238 completed). This could then be repeated in other countries to evaluate for a more global response. Another demographic issue was the lack of a centralized list of SF, as we were only able to obtain valid email addresses for 651 of the estimated 1000 SF [27], despite multiple efforts to obtain them. This lack of effort by universities to collect basic contact information about SF is also reported in the literature [37, 38] and should be addressed by administrations and departments. Despite these uncertainties regarding demographics, we found that the make-up of our SF and TF groups with respect to gender, age, and discipline did not affect the significant results in our study. Third, this study did not focus on one of the main discrepancies between SF and TF – the issue of pay and benefits. We did this purposefully in an effort to explore other motivational effects but acknowledge the impact that this discrepancy has on motivation.

More research is needed on the support needed and challenges faced by SF. FD will be more effective if the population it serves is better known so emphasis should be placed on needs assessments, utilizing focus groups to explore solutions. Further research studies should evaluate FD programs that highlight, focus on, and celebrate motivations to teach for their impact on teachers and, ultimately, on student learning.

## Conclusion

SF have become the ‘faculty majority’ and we suggest that their needs, motivations and values need to be considered in FD. The act of assessing the needs of this population, prominent in numbers but not well-represented in research, is an important first step in addressing those needs [19]. Despite some assumptions about SF lacking commitment and identity as teachers, identification of self with role/performance in teaching, altruistic professional values, and enjoyment of teaching were found to be similar between SF and TF, suggesting no difference in their motivations to be good teachers and contribute to student learning. Effective FD should not only teach skills but also reinforce and celebrate these motivations to encourage all faculty to continue to develop as educators. We suggest times for renewal and reflection on personal and professional growth [23] as well as reflection on values, reasons to teach, and the enjoyment of being a good teacher, both as individuals and as a community.

The differences between SF and TF highlight some of the issues that need to be considered when designing FD for SF. FD for SF needs to teach skills relevant to SF while promoting both motivations to teach and connectedness with the department/university. Departments need to be creative in their ways to include SF and look for ways to show appreciation for good teaching and efforts to improve. Finally, FD for SF needs to consider timing and online/hybrid formats for their offerings to accommodate SF work schedules. We suggest that assessing and addressing the motivations and needs of all faculty that have contact with students has a beneficial impact on the learning environment and the quality of education in the health sciences.

## Data Availability

The datasets used and/or analysed during the current study are available from the corresponding author on reasonable request.

## Abbreviations

AP: Motivated to try a new teaching method by forms of appreciation
CO: Perceived connectedness with their department/colleagues
FD: Faculty development
HSS: School of Health Sciences
ID: Identification with teaching
IM: Intrinsic motivation
IR: Identified regulated motivation
PMTQ: Physician Motivation Teaching Questionnaire
SDT: Self-determination theory
SF: Sessional/adjunct/contingent faculty
TF: Tenured faculty
